# Long-Distance Phasing of a Tentative “Enhancer” Single-Nucleotide Polymorphism With *CYP2D6* Star Allele Definitions

**DOI:** 10.3389/fphar.2020.00486

**Published:** 2020-05-08

**Authors:** Erin C. Boone, Wendy Y. Wang, Roger Gaedigk, Mariana Cherner, Anick Bérard, J. Steven Leeder, Neil A. Miller, Andrea Gaedigk

**Affiliations:** ^1^Division of Clinical Pharmacology, Toxicology and Therapeutic Innovation, Children's Mercy Kansas City, Kansas City, MO, United States; ^2^School of Medicine, University of Missouri-Kansas City, Kansas City, MO, United States; ^3^Department of Psychiatry, University of California, San Diego, La Jolla, CA, United States; ^4^Faculty of Pharmacy, University of Montreal, Montreal, QC, Canada; ^5^Research Center, CHU Sainte-Justine, Montreal, QC, Canada; ^6^Center for Pediatric Genomic Medicine, Children's Mercy Kansas City, Kansas City, MO, United States

**Keywords:** CYP2D6, allele definition, enhancer SNP, ddPCR = droplet digital PCR, phasing

## Abstract

**Background:**

The *CYP2D6* gene locus has been extensively studied over decades, yet a portion of variability in CYP2D6 activity cannot be explained by known sequence variations within the gene, copy number variation, or structural rearrangements. It was proposed that rs5758550, located 116 kb downstream of the *CYP2D6* gene locus, increases gene expression and thus contributes to variability in CYP2D6 activity. This finding has, however, not been validated. The purpose of the study was to address a major technological barrier, i.e., experimentally linking rs5758550, also referred to as the “enhancer” single-nucleotide polymorphism (SNP), to *CYP2D6* haplotypes >100 kb away. To overcome this challenge is essential to ultimately determine the contribution of the “enhancer” SNP to interindividual variability in CYP2D6 activity.

**Methods:**

A large ethnically mixed population sample (n=3,162) was computationally phased to determine linkage between the “enhancer” SNP and *CYP2D6* haplotypes (or star alleles). To experimentally validate predicted linkages, DropPhase2D6, a digital droplet PCR (ddPCR)-based method was developed. 10X Genomics Linked-Reads were utilized as a proof of concept.

**Results:**

Phasing predicted that the “enhancer” SNP can occur on numerous *CYP2D6* haplotypes including CYP2D6*1, *2, *5, and *41 and suggested that linkage is incomplete, i.e., a portion of these alleles do not have the “enhancer” SNP. Phasing also revealed differences among the European and African ancestry data sets regarding the proportion of alleles with and without the “enhancer” SNP. DropPhase2D6 was utilized to confirm or refute the predicted “enhancer” SNP location for individual samples, e.g., of n=3 samples genotyped as *1/*41, rs5758550 was on the *41 allele of two samples and on the *1 allele of one sample. Our findings highlight that the location of the “enhancer” SNP must not be assigned by “default.” Furthermore, linkage between the “enhancer” SNP and *CYP2D6* star allele haplotypes was confirmed with 10X Genomics technology.

**Conclusions:**

Since the “enhancer” SNP can be present on a portion of normal, decreased, or no function alleles, the phase of the “enhancer” SNP must be considered when investigating the impact of the “enhancer” SNP on CYP2D6 activity.

## Introduction

The highly polymorphic *CYP2D6* gene encodes the cytochrome P450 2D6 enzyme, which contributes to the metabolism and bioactivation of numerous clinically used drugs (Zhou, 2009a; Zhou, 2009b; Saravanakumar et al., 2019). The Clinical Pharmacogenomics Consortium (CPIC) has published guidelines for a number of *CYP2D6* gene-drug pairs (Crews et al., 2014; Hicks et al., 2015; Hicks et al., 2016; Bell et al., 2017; Goetz et al., 2018; Brown et al., 2019) substantiating the important role of CYP2D6-mediated drug metabolism. The Pharmacogene Variation Consortium (PharmVar) (Gaedigk et al., 2018b) currently defines >130 allelic variants for *CYP2D6*, which are the most important factors explaining variability in CYP2D6 activity (Hicks et al., 2013). While CYP2D6 activity is well predicted by genetic variation within the gene locus (i.e., exons and introns), there is still substantial variability among individuals with the same genotype that remains unexplained.

**Figure 1:**
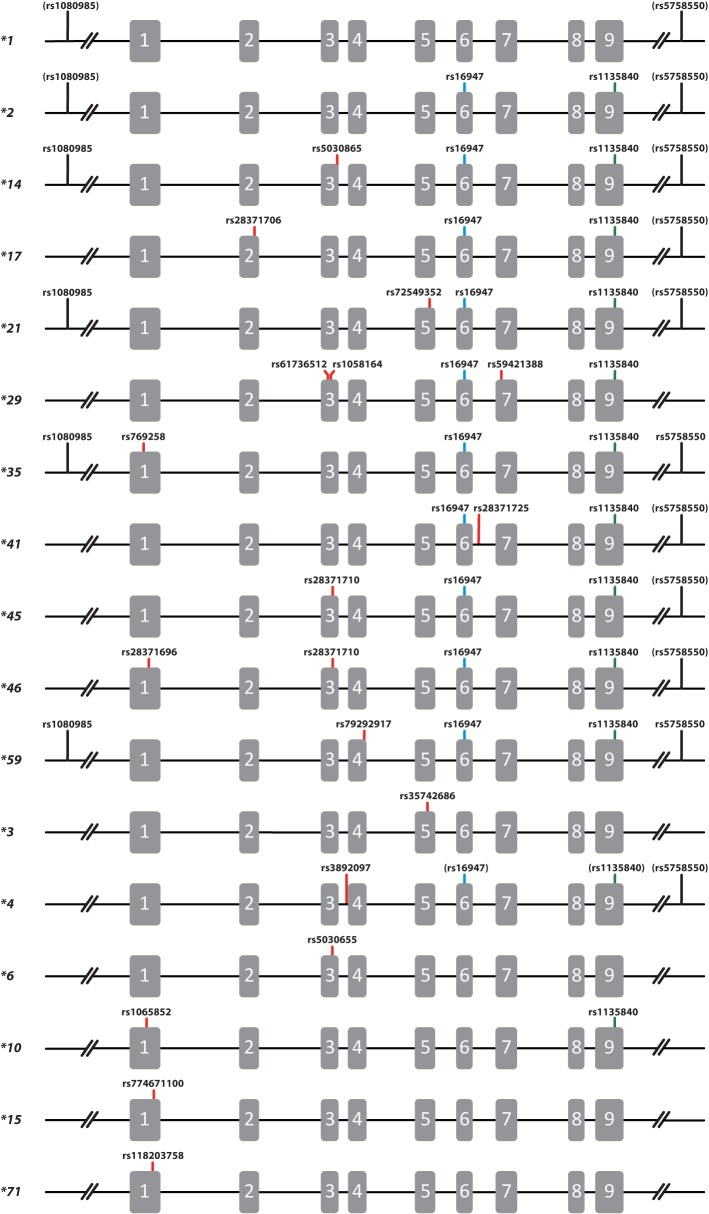
Graphical overview of *CYP2D6* alleles relevant to this study. Gray boxes represent the nine exons and lines represent intervening sequences as well as upstream and downstream regions. Each allele is displayed with its core single-nucleotide polymorphism(s) [SNP(s)], i.e., SNPs that either cause an amino acid change or impact splicing. SNPs are represented using their respective rs IDs. The SNP shown with a blue line (rs16947, g.2851C>T) highlights the CYP2D6*2 core SNP which is also part of the CYP2D6*14, *17, *21, *29, *35, *41, *45, *46 and *59 core allele definitions. The SNP shown with a green line (rs1135840, g.4181G>C) is also part of many core allele definitions. SNPs highlighted by a red line denote core SNPs rs numbers in brackets occur in some but not all alleles. In addition, the graph also shows rs1080985 (g.-1584C>G), a SNP that is most often found on CYP2D6*2 (formerly known as *2A), but has also been shown to be part of other haplotypes including CYP2D6*14, *21, *35 and *59; it is displayed in brackets if it is not present on all known suballeles. Lastly, rs5758550 denotes the “enhancer” SNP, which is shown in brackets if it does not occur on all suballeles based on the findings of this study. We refer to pharmvar.org/gene/CYP2D6 for a complete list of all SNPs found on a star allele.

CYP2D6 activity varies widely among individuals and populations (Gaedigk et al., 2017). A large portion of the observed variability can be explained by known genetic variation within the *CYP2D6* gene locus (Gaedigk et al., 2008; Gaedigk et al., 2018a; Ning et al., 2018; Dalton et al., 2019). Searching for additional loci beyond the immediate *CYP2D6* gene region, Wang et al. (2015) described that rs5758550, located 116 kb downstream of exon 9 of the *CYP2D6* gene' is associated with CYP2D6 activity. This single nucleotide polymorphism (SNP) has been proposed to impact CYP2D6 activity by modulating expression levels, and thus may account for unexplained variability, especially within a given diplotype (Wang et al., 2014; Wang et al., 2015). Wang *et al*. reported that rs5758550 “G” was associated with a two-fold increase of messenger RNA (mRNA) transcription levels (Wang et al., 2015). In a follow-up report the same group showed that 2851C>T (rs16947), which is part of the “normal function” CYP2D6*2 haplotype, was associated with a two-fold reduction in expression levels by causing alternative splicing of intron 6 (Wang et al., 2014). Taken together, the authors suggested that the presence or absence of rs5758550, which is also referred to as the *CYP2D6* “enhancer” SNP, in combination with rs16947, is a better predictor of CYP2D6 activity compared to the traditional and widely-accepted approach of using star allele-based diplotypes (or genotypes—the terms are often used interchangeably) (Wang et al., 2015; Ray et al., 2019). Based on their findings, the authors not only proposed to include rs5758550 into pharmacogenetic test panels, but also suggested that testing of rs5758550 may be more informative than testing SNPs that identify particular star alleles (Wang et al., 2014; Wang et al., 2015; Ray et al., 2019). There are, however, no additional published studies to date corroborating the impact of the “enhancer” SNP on CYP2D6 activity *in-vivo* demonstrating that combined genotyping for rs5758550 and rs16947 is superior over the current methods focusing on SNPs that identify star alleles of interest. Moreover, the “enhancer” SNP does not appear to be in complete linkage disequilibrium with rs16947, and rs16947 is not only part of the CP2D6*2 core allele definition (Gaedigk et al., 2019a), but also found on numerous other allelic variants (**Figure 1**). There are also considerable differences in the frequency of the “enhancer” SNP among populations, suggesting that this SNP may occur on many *CYP2D6* haplotypes.

The “enhancer” SNP (rs5758550) is listed by dbSNP as G>A with “G” being the minor allele with a global frequency of 28% per the gnomAD database. Wang et al. ascribed increased activity to the presence of “G” and hence referred to this nucleotide as the SNP of primary interest in their publications. Throughout this report, we refer to “G” (the “enhancer” SNP) as the variant allele and “A” as the reference allele, consistent with the dbSNP database as well as our own observations that the vast majority of CYP2D6*1 alleles have “A” at this position.

The long physical distance between the *CYP2D6* gene locus and rs5758550 poses a challenge to unequivocally determining the phase of *CYP2D6* gene locus SNPs and the “enhancer” SNP, unless both alleles carry the rs5758550 SNP. While statistical inference of linkage can be predicted by bioinformatics tools, experimental validation remains the gold standard. Methods often utilized to establish SNP linkage such as allele-specific long-range PCR or single molecule sequencing either do not allow amplification over 20 kb or are costly and labor-intensive.

In droplet digital PCR (ddPCR), a reaction is divided into up to 20,000 nanoliter-sized droplets, enabling PCR amplification from a single DNA molecule; this technology, combined with allele-specific TaqMan® probes, can rapidly phase two SNP loci that are thousands of bases apart (Roberts et al., 2014; Karlin-Neumann and Bizouarn, 2018; Lunenburg et al., 2018; Tsujimoto et al., 2018). This method is a robust and scalable molecular phasing approach that allowed Regan et al. to perform haplotype analysis of loci up to 200 kb apart (Regan et al., 2015).

This investigation aimed to address a number of questions that arose from the work published by Wang and colleagues including: what is the proportion of CYP2D6*2 alleles with and without the “enhancer” SNP? Do other haplotypes containing rs16947 also have the “enhancer” SNP—all or just some? Are there population-specific differences? In other words, to further investigate the impact of the “enhancer” SNP on CYP2D6 activity, it is essential to know which haplotypes (star alleles) can carry this SNP and experimentally establish on which allele the “enhancer” SNP is located in heterozygous samples. To provide this crucial information, we 1) computationally phased a large dataset of >3,000 ethnically diverse samples in order to determine which *CYP2D6* star alleles carry the “enhancer” SNP, and 2) established a ddPCR-based assay, referred to as the DropPhase2D6 assay, that can experimentally confirm linkage of computationally predicted haplotypes. While this investigation focuses on *CYP2D6*, it can easily be adapted to other genetic loci.

## Methods

### Study Samples

Sample and genotype data were compiled from previous studies (Abduljalil et al., 2010; Gaedigk et al., 2012; Montane Jaime et al., 2013; Tay-Sontheimer et al., 2014; Brown et al., 2016; Bérard et al., 2017; Gaedigk et al., 2019b) as well as ongoing studies (Genomic- and Ontogeny-Linked Dose Individualization and cLinical Optimization for Kids (GOLDILOKs), *CYP2D6* genotype and neurocognitive dysfunction in methamphetamine users with and without HIV and medication use in pregnant women). Studies into which participants were initially enrolled in were approved by the institutions at which the studies were performed. Written informed consent to participate in a study was provided by participants. The use of repository samples and human liver tissue samples was approved by the Children's Mercy Hospital internal review board. DNA was sourced from either whole blood, saliva, or liver tissue. The panel also included samples obtained from the Coriell Institute for Medical Research (www.coriell.org) (n=254), a PGx repository maintained at Children's Mercy (n=115), and liver tissue samples obtained from the Liver Tissue Cell Distribution System (n=150), the National Institute of Child Health and Human Development (NICHD)–supported tissue retrieval program at the University of Maryland Brain and Tissue Bank for Developmental Disorders (Baltimore, MD) (n=103) and Sekisui XenoTech LLC (n=28). Ethnicities were self-reported.

### Genotyping

Genomic DNA was prepared from whole blood or liver tissue and genotyped for a minimum of four SNPs (rs5758550 or rs133333, rs16947, rs3892097, and rs1065852) as previously described (Gaedigk et al., 1999; Gaedigk et al., 2006; Gaedigk et al., 2007; Gaedigk et al., 2008; Gaedigk et al., 2010a; Gaedigk et al., 2010b; Gaedigk et al., 2012; Gaedigk et al., 2015; Gaedigk et al., 2019b) and detailed in **Supplemental Table 1**. This format for methods reporting has been recommended by PharmVar and PharmGKB to facilitate standardized genotype method reporting (Nofziger et al., 2020). All SNP positions are in reference to the *CYP2D6* RefSeq NG_008376.3 with the ATG start codon being +1 according to PharmVar at https://www.pharmvar.org/gene/CYP2D6.

### Computational Phasing

Haplotypes were constructed from samples (n=3162) (Table 2) using PHASE (v2.1.1) (Stephens et al., 2001). Fifteen commonly genotyped SNPs were chosen for phasing analysis (**Supplemental Table 1**). An artificial “SNP” (GRCh37 chr22:42528383) was assigned as a surrogate for the CYP2D6*5 gene deletion. The majority of samples had results for all 15 SNPs and the gene deletion. Samples known to contain rare alleles (e.g., *7, *11, *21, *59, etc.) were excluded from the computational data analysis. Ethnicity was available for n=2,564 samples. Phasing was performed for all samples (n=3162), regardless of ethnicity (dataset_all). Separate population-specific analyses were performed for samples with African (n=603) and European (n=1474) ancestries (dataset_A and dataset_E).

### DNA Preparation

Long-distance phasing by ddPCR requires high molecular weight (HMW) DNA samples. To assess the suitability of DNA for DropPhase2D6, different commercially available kits were evaluated. To limit shearing, all pipetting steps (including handling of blood and DNA) were carried out with 200 µl BioClean Ultra™ Wide-O LR Filter Sterilized pipette tips (Rainin, Oakland, CA) and 10 µl BioClean Ultra™ Filter tips cut with a scalpel to create a wide bore tip. Ethylene-diaminetetraacetic acid (EDTA) whole blood samples were processed and frozen at −80°C within 24 hrs of collection. White blood cells were isolated from blood, resuspended in 1x phosphate-buffered saline (2.7 mM KCl, 10 mM KH_2_PO_4_, 137 mM NaCl, 1.8 mM anhydrous Na_2_HPO_4_, pH 7.4) in 20% of the original blood volume, and immediately used for DNA extraction or frozen at −80°C.

DNA quality was assessed by 0.5% agarose gel electrophoresis and the concentration determined using a NanoDrop One instrument (Thermo Fisher Scientific, Waltham, MA).

#### DNeasy Blood and Tissue Kit (Qiagen, Hilden, Germany)

To serve as a comparison to HMW DNA preparations, DNA was isolated with this silica column-based DNA extraction kit. Blood and tissue samples were prepared as per manufacturer's protocol.

#### PrepFiler™ Forensic DNA Extraction Kit (Applied Biosystems, Foster City, CA)

DNA was extracted from whole blood or white blood cells with the PrepFiler Forensic DNA Extraction Kit as described by Regan et al. (2015) with the following modifications: 200 µl of frozen whole blood or 40 µl of white blood cell suspension were used in lieu of cultured cells. The elution step was repeated 3x, as subsequent elutions from the beads yielded increasing quality of HMW DNA.

#### MagAttract^®^ HMW DNA Kit (Qiagen, Hilden, Germany)

DNA was extracted from whole blood, white blood cell suspensions or 25 mg of frozen liver tissue per manufacturer's protocol with the following modifications: 50 µl of suspended white blood cells or 50 µl of whole blood was used instead of 200 µl whole blood and the tubes were gently flicked instead of shaken at 1,400 rpm.

#### MegaLong™ DNA Extraction Kit (G-Biosciences, St. Louis, MO)

DNA was extracted from whole blood, white blood cell suspension or 25 mg of frozen liver tissue per manufacturer's protocol with the following modifications: 100 µl of frozen whole blood or 15 µl of white blood cell suspension was substituted for fresh whole blood. Cell suspensions were centrifuged at 16,000xg for 10 min, instead of 5 min, to pellet the cells and then washed with 100 µl of the nuclei isolation buffer after removing the supernatant. This additional wash step facilitated the removal of excess lysis debris.

### Experimental Phasing Using ddPCR—DropPhase2D6

#### Probe and Assay Design

TaqMan® SNP assays were custom ordered from Life Technologies (Carlsbad, CA, USA). Each 40x assay consisted of two probes, one with a fluorophore and quencher while the other had only a non-florescent quencher (NFQ), referred to in this report as a “dark probe.” For 2851C>T (rs16947), two 40x assays were designed, i.e., T-FAM+C-dark and C-FAM+T-dark, allowing us to detect signal for either the “C” or “T” allele. For all other assays, VIC labeled probes were used. Another probe set for the CYP2D6*4 core SNP (1847G>A) was validated after the method was established for 2851C>T. DropPhase2D6 assay names, their TaqMan® assay IDs, and the SNP-specific probes labeled with fluorophores are shown in **Table 1**.

**Table 1:** Custom Dark Probe TaqMan® assays for DropPhase2D6.

TaqMan® assay ID	DropPhase2D6 assay name	Custom ID	rs number	SNP location	Dye	Fluorophore on	NFQ on
C__27102425_10	rs16947varT	C__27102425_10AF	rs16947	exon 6	FAM	A	G
C__27102425_10	rs16947refC	C__27102425_10GF	rs16947	exon 6	FAM	G	A
C__27102431_D0	rs3892097varT	C__27102431_D0TF	rs3892097	intron 3	FAM	C	T
C__61226317_10	rs28817600varG	C__61226317_10GV	rs28817600	25 kb	VIC	G	A
C__30485108_10	rs5758562varC	C__30485108_10CV	rs5758562	75 kb	VIC	C	G
C__29692254_10	rs5758550varG	C__29692254_10GV	rs5758550	116 kb	VIC	G	A
C__29692254_10	rs5758550refA	C__29692254_10AV	rs5758550	116 kb	VIC	A	G

TaqMan® Assay ID and Custom Assay ID refer to the original TaqMan® assays and their corresponding customized assay. The fluorescent dye (FAM and VIC) are as specified. For example, C__27102425_10AF is a modified C__27102425_10 assay allowing interrogation of rs16947 “A” with FAM. In the text we refer to the assays by their DropPhase2D6 assay name, which includes the SNP rs number and the allele of interest (generating a fluorescent signal). The SNP location indicates the location of rs16947 and rs3892097 within the CYP2D6 gene and the approximate downstream positions of SNPs interrogated for linkage with rs16947. NFQ, non-fluorescent quencher (or dark probe). Note that dbSNP reports rs5758562C>G (MAF=0.27) while we refer to “G” as reference and “C” as the variant.

#### Linkage Duplex Assays

For each linkage reaction, two 40x assays were combined: an rs16947-FAM assay with a VIC-containing assay. For example, to evaluate linkage between rs16947 “T” and rs5758550 “G,” the rs16947varT and rs5758550varG assays were used. We will refer to the combination of the two custom TaqMan® SNP assays as a “duplex.” DropPhase2D6 was carried out as follows: 1) the two selected 40x assay mixes were combined with ddPCR Supermix for Probes without dUTP (Bio-Rad, Hercules, CA) and added to each well; 2) 10–30 ng HMW DNA was added; 3) H_2_O was added for a total volume of 22 µl, and 4) the reaction was gently mixed by pipetting 15 times with a Rainin wide-orifice P200 tip; 5) the 96 well plate was gently vortexed at low speed for three pulses and spun for 2 min at 2,000xg. After generating droplets using the Auto Droplet Generator (Bio-Rad, Hercules, CA), the plates were heat-sealed with a foil cover (Bio-Rad) and cycled in a C1000 Touch Thermocycler (Bio-Rad). The thermal cycling protocol was as follows: 10 min at 95°C for 1 cycle, 40 cycles of 30 s at 94°C and 1 min at 60°C, and a final cycle of 98°C for 10 min. Droplets were read on the QX-200 Droplet Digital PCR system (Bio-Rad) and linkage analysis was performed with the QuantaSoft™ Analysis Pro software v.1.0.596. Linkage is expressed as a percent of all molecules that are linked as described in Regan et al. (2015).

Two control strategies were utilized for the DropPhase2D6 experiment. First, 150 ng of HMW DNA was digested with 10 U of *Eco*RI-HF restriction enzyme (RE) (New England Biolabs, Ipswich, MA) at 37°C for 1.5 h followed by RE inactivation at 65°C for 20 min. Digested DNA was subsequently used in place of HMW DNA as a no-linkage control. The second strategy included testing all possible allelic configurations of a compound heterozygote. This is achieved by performing two duplex reactions. One duplex reaction is expected to be in linkage (interrogated SNPs are in *cis*) while the other duplex reaction is expected not to show linkage (interrogated SNPs are in *trans*). For example, in a sample genotyped as CYP2D6*1/*2 (heterozygous for rs16947 and rs5758550), linkage between the interrogated SNPs is observed when using the rs16947varT+rs5758550varG duplex reaction, while the rs16947refC+rs5758550varG duplex reaction results in no linkage. If linkage cannot be established with one of the duplex reactions, DNA quality is likely insufficient.

To determine linkage, we adopted a conservative cut-off value of 5% linked molecules, i.e., linkage must be greater than 5% for SNPs over 100 kb apart to be called as linked. Additionally, the difference between the positive and negative control duplex reactions must have at least a 5% linked molecules difference. For example, interrogated SNPs are determined to be linked, if the rs16947varT+rs5758550varG duplex reaction yields e.g., 10% linked molecules and the rs16947refC+rs5758550varG duplex reaction yields 0.3% linked molecules and thus differ by >5% between the two reactions. The SNPs are not called linked if the duplex reaction yields e.g., 4.5% linked molecules and the rs16947refC+rs5758550varG duplex reaction yields 0.5% linked molecules.

Assay conditions linking the “enhancer” SNP with 1847G>A were identical to those described for 2851C>T.

#### Long Distance Phasing With 10x Genomics

The 10X Genomics Linked-Reads platform allows phasing of sequence variations across >10 Mb haplotype blocks ( https://www.10xgenomics.com). Publicly available 10X Genomics Linked-Read data from the Illumina HiSeqX-PGx Cohort were used as proof-of-concept for phasing the “enhancer” SNP with *CYP2D6* haplotypes, i.e., selected star allele-identifying core SNPs. Data were obtained at https://github.com/Illumina/Polaris/wiki/HiSeqX-PGx-Cohort and analyzed with Long Ranger v2.2.2 and Loupe software v2.1 (10XGenomics, Pleasanton, CA) against the GRCh37 reference genome.

## Results

### Computational Phasing

In order to determine which *CYP2D6* haplotypes (or star alleles) carry the “enhancer” SNP, a dataset comprising genotype information from 3,162 samples (based on 15 SNPs and a surrogate “SNP” representing the CYP2D6*5 gene deletion) was subjected to computational haplotype phasing. Phasing was performed on all samples (dataset_all) and by ethnicity/race (n=1482), European (White, Caucasian; dataset_E), and n=609, African (Black, Africa, African American; dataset_A). Haplotype predictions and frequencies for all three datasets are summarized in **Table 2**. As shown in this table, predictions varied considerably for CYP2D6*5, *29, and *41 haplotypes, depending on whether phasing was performed on the entire multiethnic cohort or within their racial/ethnic group. For example, separately phasing the European and African datasets suggested that only one of the alleles phased as CYP2D6*29 has the “enhancer” SNP. In contrast, when phasing was done on the entire cohort, 16 alleles were predicted to have the “enhancer” SNP on a CYP2D6*29 allele. However, as described below, experimental phasing with DropPhase2D6 ultimately demonstrated that the “enhancer” SNP was not located on the CYP2D6*29 allele in this sample.

**Table 2:** Computational haplotype determination using PHASE.

Star allele	Allele carries	Haplotype frequency dataset_all n=6,324	Haplotype frequency dataset_E n=2,964	Haplotype frequency dataset_A n=1,218	Haplotype frequency dataset_E n=2,964	Haplotype frequency dataset_A n=1,218
		All samples phased together; n=number of alleles			Phased E only	Phased A only
		n= Number of allele (% of given allele)				
*1	none	2,236 (94.6%)	1,104 (98.6%)	304 (76.9%)	1,110 (99.1%)	280 (70.7%)
	rs5758550 (enhancer SNP)	121 (5.1%)	12 (1%)	91 (23%)	6 (0.5%)	116 (29.2%)
	rs1080985 (−1584C>G)+ rs5758550 (enhancer SNP)	3 (0.1%)	2 (0.1%)	0 (0%)	3 (0.2%)	0 (0%)
	rs1080985 (−1584C>G)	2 (0%)	1 (0%)	0 (0%)	0 (0%)	0 (0%)
*10	none	248 (98.8%)	48 (97.9%)	43 (97.7%)	48 (97.9%)	42 (95.4%)
	rs5758550 (enhancer SNP)	3 (1.1%)	1 (2%)	1 (2.2%)	1 (2%)	1 (2.2%)
	rs5030655 (*6)	0 (0%)	0 (0%)	0 (0%)	0 (0%)	1 (2.2%)
*17	none	4 (1.6%)	0 (0%)	3 (1.5%)	0 (0%)	5 (2.5%)
	rs5758550 (enhancer SNP)	236 (98.3%)	13 (100%)	194 (98.4%)	13 (100%)	192 (97.4%)
*2	none	66 (6%)	3 (0.5%)	60 (27.6%)	4 (0.7%)	63 (28.6%)
	rs1080985 (−1584C>G)	33 (3%)	18 (3.5%)	2 (0.9%)	17 (3.3%)	0 (0%)
	rs5758550 (enhancer SNP)	94 (8.6%)	11 (2.1%)	65 (29.9%)	11 (2.1%)	65 (29.5%)
	rs1080985 (−1584C>G)+ rs5758550 (enhancer SNP)	898 (82.3%)	476 (93.7%)	90 (41.4%)	477 (93.7%)	92 (41.8%)
*29	none	113 (87.5%)	3 (100%)	94 (87%)	2 (100%)	106 (99%)
	rs5758550 (enhancer SNP)	15 (11.6%)	0 (0%)	14 (12.9%)	0 (0%)	1 (0.9%)
	rs1080985 (−1584C>G)+ rs5758550 (enhancer SNP)	1 (0.7%)	0 (0%)	0 (0%)	0 (0%)	0 (0%)
*3	none	66 (100%)	48 (100%)	4 (100%)	48 (100%)	4 (100%)
*35	rs1080985 (−1584C>G)	7 (2.9%)	7 (4.3%)	0 (0%)	9 (5.5%)	0 (0%)
	rs1080985 (−1584C>G)+ rs5758550 (enhancer SNP)	229 (97%)	154 (95.6%)	4 (100%)	152 (94.4%)	4 (100%)
*4	none	974 (99.2%)	561 (99.1%)	95 (100%)	561 (99.1%)	95 (100%)
	rs5758550 (enhancer SNP)	1 (0.1%)	1 (0.1%)	0 (0%)	1 (0.1%)	0 (0%)
*4.012	none	6 (0.6%)	4 (0.7%)	0 (0%)	4 (0.7%)	0 (0%)
*40	none	12 (100%)	0 (0%)	10 (100%)	0 (0%)	10 (100%)
*41	none	489 (97.9%)	282 (97.9%)	32 (94.1%)	276 (95.8%)	30 (96.7%)
	rs5758550 (enhancer SNP)	9 (1.8%)	5 (1.7%)	2 (5.8%)	11 (3.8%)	1 (3.2%)
	rs1080985 (−1584C>G)+ rs5758550 (enhancer SNP)	1 (0.2%)	1 (0.3%)	0 (0%)	1 (0.3%)	0 (0%)
*45	none	56 (98.2%)	1 (100%)	41 (97.6%)	1 (100%)	41 (97.6%)
	rs5758550 (enhancer SNP)	1 (1.7%)	0 (0%)	1 (2.3%)	0 (0%)	1 (2.3%)
*5	none	199 (85%)	88 (90.7%)	45 (72.5%)	88 (90.7%)	56 (90.3%)
	rs5758550 (enhancer SNP)	35 (14.9%)	9 (9.2%)	17 (27.4%)	9 (9.2%)	6 (9.6%)
*6	none	39 (100%)	32 (100%)	1 (100%)	32 (100%)	1 (100%)
*69	none	1 (100%)	0 (0%)	0 (0%)	0 (0%)	0 (0%)
*9	none	126 (100%)	79 (100%)	5 (100%)	79 (100%)	5 (100%)

Phasing was performed using dataset_all (all samples, n=3,162) and frequencies for samples with European and African ancestry calculated after phasing was performed. Phasing for dataset_E (samples with European ancestry, n=1,482) and dataset_A (samples with African ancestry, n=609) was performed on each dataset separately. Frequency denotes the frequency of the haplotype per PHASE output. The left-hand column provides the core star allele designation to which a haplotype matches. *4.012 denotes a *4 suballele lacking 100C>T, a SNP that is present in all other defined *4 suballeles. Of note, dbSNP reports rs1080985 as G>C (gnomAD MAF=0.2) while we refer to “C” as reference and “G” as variant. none denotes that allele does not have rs5758550 ("enhancer" SNP) or rs1080985 (-1584C>G).

Phasing revealed that the “enhancer” SNP is most often found on haplotypes carrying the CYP2D6*2 core SNP (2851C>T, rs16947) including CYP2D6*2, *17, and *35 among others (**Figure 1**; for core allele definitions see Gaedigk et al., 2019a; Nofziger et al., 2020). However, regardless of ethnicity, star allele haplotypes were not in complete linkage with the “enhancer” SNP. For example, while almost all CYP2D6*2 in the European subset were predicted to carry rs5758550 “G” (95.8%), only 71.3% of samples with African ancestry were predicted to carry it (**Table 2**). Phasing also suggested that a substantial proportion of CYP2D6*1 alleles carry the “enhancer” SNP (0.7 and 29.2%, respectively, when European and African ancestry samples were phased separately), a finding that needs to be viewed with caution. For example, since CYP2D6*1 is a default assignment (e.g., *15, *22–*27, *33, *39, *43 and others were not discriminated by genotyping and therefore were designated *1 by “default”) the number of true *1 alleles carrying the “enhancer” SNP may be lower. Similarly, CYP2D6*11, *12, *19–*21, *59, and numerous other haplotypes were defaulted to a CYP2D6*2 assignment which likely contributed to an over-estimation of true *2 alleles carrying the “enhancer” SNP.

Another SNP of interest was −1584C>G (rs1080985) which in the past has been implicated to play a regulatory role for CYP2D6 function (Raimundo et al., 2000; Raimundo et al., 2004). This SNP is predominantly, but not exclusively, found on CYP2D6*2 alleles. As shown in **Table 2**, rs1080985 (−1584C>G) and rs5758550 (“enhancer” SNP) are in linkage disequilibrium, i.e., the majority of CYP2D6*1 alleles do not have either SNP, while the majority of CYP2D6*2 alleles possess both. This table provides predicted frequencies stratified by allele and the presence of −1584C>G (rs1080985) and/or rs5758550 (“enhancer” SNP). For example, within dataset_E (Europeans) 509 alleles were called CYP2D6*2 by the PHASE algorithm of which four (0.7%) were predicted to not have −1584C>G or the “enhancer” SNP, 17 (3.3%) were predicted to have −1584C>G, 11 (2.1%) were predicted to have the “enhancer” SNP, and 477 (93.7%) were predicted to have both SNPs. It follows that the cumulative predicted frequency of CYP2D6*2 alleles with the “enhancer” SNP is 95.8%.

For other ethnic groups it was also observed that the majority of CYP2D6*2 alleles carry the “enhancer” SNP as well as the SNP at position -1584. Hispanics (n=176 subjects), East Asians (n=98 subjects), and subjects of Indian ancestry (n=210), 69/352, 26/196, and 77/410 alleles were phased as CYP2D6*2 with 62 (90%), 18 (69%), and 74 (96%), respectively and predicted to carry both SNPs. In African Americans, however, only 42% of CYP2D6*2 alleles are computationally predicted to have the “enhancer” SNP as well as −1584C>G. Differences were also observed for other alleles between these populations and our larger European and African-ancestry cohorts (not shown). These predictions are, however, estimates due to the small numbers of alleles observed with or without the “enhancer” SNP and HMW DNA was not available for experimental confirmation.

### DropPhase2D6 Assay Validation

**Figure 2:**
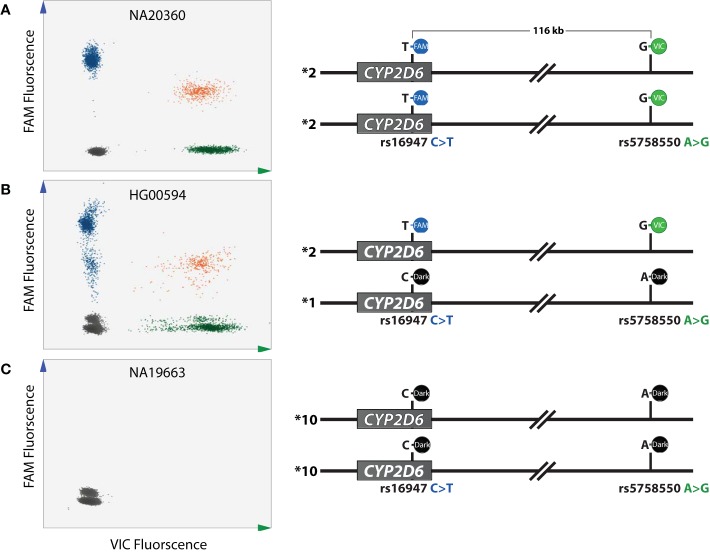
DropPhase2D6 assay development. Assays designed for DropPhase2D6 were evaluated using Coriell DNA samples with known genotypes. **(A)** NA20360 is homozygous variant for the two single-nucleotide polymorphisms (SNPs) of interest yielding three distinct clusters: green, VIC [droplets containing genomic DNA (gDNA) with rs5758550G)], blue FAM (droplets containing gDNA with rs16947T), and orange, denoting a double-positive cluster (droplets containing DNA molecules generating FAM and VIC signal). **(B)** HG00594 is heterozygous for both SNPs displaying the same cluster pattern as shown in **(A)**. The observed “rain” on the scatter plot is a commonly observed feature believed to be due to probe competition for PCR reaction components. **(C)** NA19663 is homozygous reference for both loci, therefore no fluorescent signals are produced (no green, blue or orange clusters). A secondary cluster above the gray double-negative cluster was observed for HG00594 **(B)** and NA19663 **(C)** which is likely due to residual signal originating from the rs16947T-labeled binding to the rs16947C allele. This phenomenon is absent in the homozygous variant sample NA20360, which lacks rs16947C.

To establish DropPhase2D6, assays were tested using six Coriell DNA samples with known genotypes for rs5758550 (“enhancer” SNP) and rs16947 (**Figure 2**): NA19663, HG00650 (homozygous reference), HG00594, NA12248 (compound heterozygote), and HG00275, NA20360 (homozygous variant).

When interrogating NA20360 (homozygous variant), using the rs16947varT+rs5758550varG duplex reaction (**Table 1**), FAM as well as VIC signals were detected. Analysis with QuantaSoft software displayed four different clusters, shown in **Figure 2A**: the single fluorophore-positive clusters (blue and green) represent signals produced from droplets containing DNA fragments harboring one of the two interrogated SNPs (DNA breaks may have occurred between the two SNPs “unlinking” signals into discrete clusters); a cluster that is positive for both fluorophores (orange), representing droplets containing DNA fragments carrying both SNPs or, in rare cases, multiple (often smaller) DNA fragments that were partitioned into the same droplet by chance, and the cluster with no signals (gray) representing droplets that do not contain DNA with either of the interrogated SNPs. Note that cluster density is inversely correlated with DNA integrity, i.e., the intensity of the blue and green clusters is lower for samples with higher integrity DNA compared to samples with lower-integrity DNA. We also observed a lower signal height of the double-positive FAM and VIC cluster (orange) compared to the single positive FAM (blue) or VIC (green) clusters which we attributed to decreased PCR efficiency when both are present in the same droplet. As described by Whale et al., this does not interfere with cluster interpretation as long as the double-positive cluster is distinct and identified by the QuantaSoft software (Whale et al., 2016).

When both loci are heterozygous (illustrated for HG00594 in **Figure 2B**), three positive fluorophore clusters were observed. Note that the reference alleles in this assay are not visualized on the scatter plot as they are targeted by “dark” probes. However, when both the reference and variant alleles are present in a droplet, fluorescent hydrolysis endpoint may not be achieved because of competition for the reaction components. As a result, there are droplets generating varying fluorescent intensities which presented as “rain” on the scatter plot (Regan et al., 2015). For HG00594 we also observed a low amplitude gray FAM sub-cluster, near the no-fluorophore cluster. NA19663 (homozygous reference for rs16947 and rs5758550) also presented with this low amplitude sub-cluster (**Figure 2C**). The presence of this cluster in both the homozygous reference and the compound heterozygous control samples and not in the homozygous variant control, is likely caused by unspecific binding of the rs16947varT probe to the rs16947 “C” allele. The signal of this sub-cluster is automatically combined with the no-fluorophore cluster by the QuantaSoft software and therefore does not interfere with cluster analysis. Interference by the *CYP2D7* gene is unlikely since no other cluster(s) were present in NA19663 (or other samples with the same genotype) (**Figures 2B, C**).

Taken together, we demonstrated that the custom TaqMan® assay reaction set-up can determine SNP linkage over a distance of 116 kb.

### DropPhase Mile Marker

**Figure 3:**
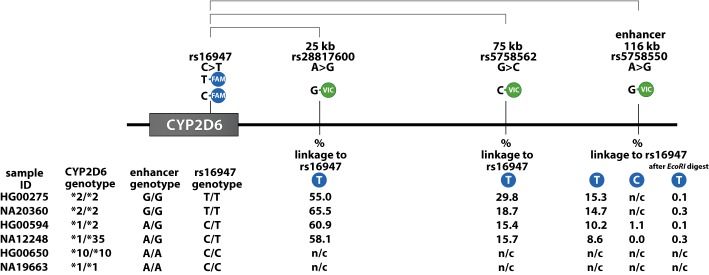
Mile Post experiment to establish single-nucleotide polymorphism (SNP) linkage. SNPs at increased distances relative to the CYP2D6*2 core SNP (rs16947) were interrogated to establish DropPhase2D6. The percent (%) linkage between SNPs is decreasing as the distance between interrogated SNPs increases. SNPs that are *trans*-configured show no/little linkage (e.g., rs16947A and rs5758550G). No linkage was observed when DNA was pre-treated with the restriction enzyme *Eco*RI. n/c, negative control, i.e., genotype does not support signal generation with respective probe/assay combinations.

A “mile marker” experiment was utilized to establish linkage between rs16947 within the *CYP2D6* gene and selected SNPs at distances of 25 kb (rs28817600 “G”), 75 kb (rs5758562 “C”), and the “enhancer” SNP at over 116 kb (rs5758550 “G”) (**Table 1**, **Figure 3**). As expected, with increased distances between the interrogated SNPs, decreased percentages of linked molecules were observed, which we attributed to DNA integrity. This experimental set-up allowed us to evaluate the suitability of DNA preparations for long-distance phasing.

To avoid “artificial linkage” signals that may be caused by uneven distribution of DNA during droplet generation, two different negative control reactions were performed (**Figure 3**): 1) HMW DNA was subjected to the restriction enzyme *Eco*RI, that cuts DNA between the SNPs of interest, and 2) the FAM-labeled assay for rs16947 “C” (reference) was paired with the VIC-labeled assay for rs5758550 “G” (variant). This duplex reaction served as a negative control, while the rs16947varT+rs5758550varG duplex reaction detected linkage for sample HG00594. Furthermore, no linkage signal is observed for the rs16947varT+rs5758550varG duplex reaction when the DNA was pretreated with *Eco*RI. Thus, the combined results of these two duplex assays corroborate that rs16947 “T” and rs5758550 “G” are *cis*-configured, while rs16947 “C” and rs5758550 “G” are *trans*-configured (**Figure 3**).

### High Molecular Weight DNA Isolation Methods

Since silica column-based DNA extraction methods, including the DNeasy Blood and Tissue Kit, did not produce DNA of sufficient integrity for long range experimental phasing, we therefore evaluated different methods to obtain HMW DNA from blood and liver tissue samples (**Table 3**). DNA isolated from whole blood or white blood cells with the PrepFiler Forensic DNA Extraction kit supported linking SNPs at distances of 25 kb and 116 kb, but were not completely abolished when the DNA was pretreated with *Eco*RI (to serve as a negative control) (**Table 3**). In addition, we were unable to extract DNA of sufficient integrity from frozen liver tissue (assessed by gel electrophoresis) to perform DropPhase2D6 (not shown).

**Table 3:** DropPhase2D6 using high molecular weight (HMW) DNA prepared with different methods.

DNA Kit	Source	*CYP2D6* Genotype	rs16947varT + rs28817600varG 25 kb	rs16947varT + rs28817600varG (+RE) 25 kb	rs16947varT + rs5758550varG 116 kb	rs16947varT + rs5758550varG (+RE) 116 kb	rs16947refC + rs5758550varG 116 kb
DNeasy	WB	*1/*2	15.45	0.33	0.64	0.34	0.03
DNeasy	Tissue	*1/*2	1.8	0.14	-0.66	ND	-0.39
PrepFiler Forensic	WB	*1/*2	49.06	16.36	4.69	2.93	0.8
PrepFiler Forensic	WBC	*1/*2	57.26	18.85	**6.85**	**1.72**	**0.62**
MagAttract	WB	*1/*17	52.84	0.64	3.83	0.71	−0.38
MagAttract	WBC	*1/*2	50.18	−1.1	1.44	2.47	−0.8
MagAttract	Tissue	*1/*2	25.81	−0.4	0.48	0.09	0.44
MegaLong	WB	*1/*2	82.84	3.13	**40.21**	−**0.01**	−**2.58**
MegaLong	WBC	*2/*4	66.43	2.49	**41.72**	−**2.41**	−**0.02**
MegaLong	Tissue	*1/*2	63.9	3.89	**13.62**	**0.73**	**1.16**

DNA was prepared from liver tissue, whole blood (WB), and white blood cells (WBC) using different commercially available DNA extraction kits. All kits produced DNA of sufficient integrity to perform linkage over a distance of 25 kb. The MegaLong kit was, however, superior for phasing over the longer distance of 116 kb, as demonstrated by high values of percent linkage. For DNA samples prepared with the PrepFiler Kit, false-positive linkage was observed in the negative controls reactions, i.e., when DNA was pre-treated with restriction enzyme EcoRI (+RE). This was only observed for this extraction kit and is likely due to incomplete digestion by the enzyme. Samples with at least 5% linked molecules and a difference of at least 5% linked molecules between positive and negative control duplexes are shown in bold.

The linkage results for DNA extracted with the Qiagen MagAttract kit were comparable to those described for the PrepFiler Forensic Kit. The integrity of DNA extracted from liver tissue was also not sufficient. In contrast to the PrepFiler Forensic Kit, *Eco*RI*-*pretreated DNA was digested to completion and did not yield linkage rates above background (**Table 3**).

The dialysis-based G-Biosciences MegaLong kit was the only commercial kit that yielded the long fragments of DNA necessary for DropPhase2D6 from whole blood, white blood cells, and liver tissue. The DNA prepared with this kit could also be readily cut with the *Eco*RI restriction enzyme resulting in minimum residual linkage. Also, DNA extracted with the MegaLong kit produced the overall highest linkage percentages of all methods evaluated.

Because the G-Biosciences MegaLong Kit yielded superior HMW DNA preparations across all sample sources and produced consistent linkage results for SNPs at 25 kb and 116 kb, this kit was employed to prepare all subsequent samples.

### Experimental Phasing With DropPhase2D6

**Figure 4:**
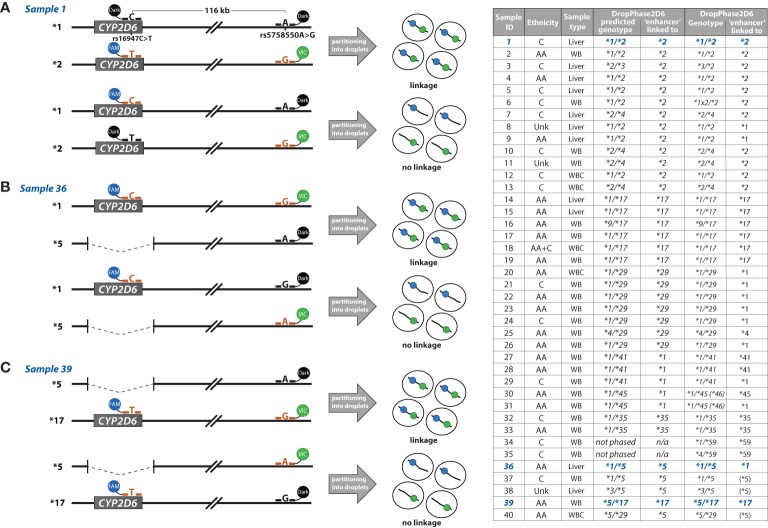
DropPhase2D6 summary and data. The figure provides DropPhase2D6 results for selected samples, sample types and duplex reactions used to experimentally link the “enhancer” single-nucleotide polymorphism (SNP) with *CYP2D6* haplotype. Phase-predicted genotypes were compiled from datasets_all (all ethnicities phased together). **(A)** Sample 1 was genotyped as CYP2D6*1/*2.001. The “enhancer” SNP was confirmed to be *cis*-configured, i.e., located on the *2.001 allele. The rs16947varT+rs5758550varG and rs16947refC+rs5758550varG duplex reactions were utilized to establish linkage. **(B)** Sample 36 was genotyped as CYP2D6*1/*5. The “enhancer” SNP was confirmed to be *cis*-configured, i.e., located on the *1 allele as predicted by computational phasing. The rs16947refC+rs5758550varG and rs16947refC+rs5758550refA duplex reactions were utilized to establish linkage for this case. **(C)** Sample 39 was genotyped as CYP2D6*5/*17. The duplex reactions rs16947varT+rs5758550varG and rs16947varT+rs5758550refA were used to establish linkage. The “enhancer” SNP was confirmed to be *cis*-configured, i.e., located on the CYP2D6*17 allele as predicted by computational phasing. The middle panel visualizes DNA molecules sequestered in droplets with colors representing SNPs generating VIC (green) or FAM (blue) signals. The right-hand table provides sample source (liver tissue; WB, whole blood; WBC, white blood cells), genotype and ethnicity **(C)**, Caucasian/European ancestry, AA (African ancestry) Unk (unknown), and AA+C (mixed ancestry). The far-right column indicates to which allele the “enhancer” SNP was experimentally linked. DropPhase2D6 results for samples 11, 25 and 35 were confirmed with the assay for the CYP2D6*4 core SNP. Specifically, the reference allele of the “enhancer” SNP was linked with 1847G (rs3892097. reference G) for samples 11 and 35, while linkage between the “enhancer” SNP and 1847A (rs3892097, variant A) was confirmed for sample 25.

Of the DNA samples available for further study, n=40 samples were chosen for DropPhase2D6 based on the following criteria: availability of blood or tissue for HMW DNA preparation, genotype (heterozygous for rs5758550 and rs16947), ambiguous phase call and/or was genotyped for a rare allele e.g., CYP2D6*59 which was not captured by computational phasing. As detailed in **Figure 4**, DropPhase2D6 was performed with the rs16947varT+rs5758550varG and rs16947refC+rs5758550varG duplex reactions. **Figure 4A** displays the result for a sample with a CYP2D6*1/*2 genotype for which DropPhase2D6 demonstrated that the “enhancer” SNP is located on the *2 allele. **Figure 4B** provides an example of a sample with a CYP2D6*1/*5 genotype. In this case, DropPhase2D6 was performed with the rs16947refC+rs5758550varG and rs16947refC+rs5758550refA duplex reactions which revealed that the “enhancer” SNP is in *cis* with the CYP2D6*1 haplotype. **Figure 4C** provides the result for a sample genotyped as CYP2D6*5/*17 tested with the rs16947varT+rs5758550varG and rs16947varT+rs5758550refA duplex reactions. In this example, the “enhancer” SNP was linked with rs16947, which is part of the CYP2D6*17 haplotype (**Figure 1**). The table in **Figure 4** provides the results, along other pertinent information for the 40 samples selected for experimental validation by DropPhase2D6. The presence of the “enhancer” SNP was confirmed for the majority of CYP2D6*2 alleles, but also demonstrated that the “enhancer” SNP can be located on other haplotypes including CYP2D6*1, *4, *5, *17, *35, *41, *45, and *59. We did not find the “enhancer” SNP on any CYP2D6*3, *9, or *29 alleles. HMW DNA was not available for the two samples for which PHASE predicted the “enhancer” SNP to be on the CYP2D6*10 allele. For the sample genotyped as CYP2D6*4/*29 and predicted by PHASE to have the “enhancer” SNP on the *29 allele, DropPhase2D6 revealed that rs16947T, which is part of the*29 haplotype, is in *trans* with the “enhancer” SNP indicating that it is on the *4 allele. The location of the “enhancer” SNP on the CYP2D6*4 allele was confirmed by finding 1847A *in cis* with the “enhancer” SNP (**Figure 4**). The 1847G>A assay allows to experimentally phase the “enhancer” SNPs for samples with genotypes such as CYP2D6*1/*4.

Eleven Coriell DNA samples for which 10X Linked-Reads were available were chosen for experimental confirmation by DropPhase2D6. Unfortunately, only sample HG00589 was of sufficient integrity to determine linkage (i.e., linkage was >5%) (**Table 4**). An additional five Coriell samples were tested with DropPhase2D6; these were previously predicted by Ray et al. (2019) to either harbor novel *CYP2D6* haplotypes or have unique haplotypes regarding the “enhancer” SNP (Coriell IDs were kindly provided by Danxin Wang) (**Table 5**). All five samples met the 5% threshold and therefore allowed us to successfully determine the phase of the “enhancer” SNP. The haplotypes provided by Ray et al. were found to be inconsistent with our genotyping results and thus, the “enhancer” SNP was not detected on a rare allele as predicted (**Table 5**). For example, HG00190 was genotyped as CYP2D6*1/*2 and the “enhancer” SNP linked with the *2 allele which contrasts the “*3 with “enhancer” SNP” prediction by Ray et al. (2019). Another example is NA19338 for which Ray et al. predicted that “*2 and *9 SNPs are on the same allele”. However, this sample was genotyped as CYP2D6*2/*5 with DropPhase2D6 linking the “enhancer” SNP to the *5 allele.

**Table 4:** 10X Genomics Linked-Reads technology to phase enhancer single-nucleotide polymorphism (SNP).

Sample ID	Genotype	Allele predicted to carry the “enhancer” SNP per PHASE	Allele to which the “enhancer” SNP is linked by 10X Linked-Reads
HG00436	*2x2/*71	N/A^1^	*2
HG00589	*1/*21	N/A^1^	*21^2^
NA12003	*2/*3	*2	*2
NA12813	*2/*4	*2	*2
NA18552	*1/*14	N/A^1^	*14
NA18959	*2/*36+*10	*2	*2
NA18973	*1/*21	N/A^1^	*21
NA18980	*2/*36+*10	*2	*2
NA19207	*2/*10	*2	*2
NA19239	*15/*17	N/A^1^	*17
NA19819	*2/*4x2	*2	*2

DNA samples investigated for linkage between rs16947 and rs5758550 using 10X Genomics Linked-Reads. Only one sample, HG00589, was of sufficient integrity to support DropPhase2D6 (visualization see **Supplemental Figure 1**). ^1^Samples contained rare CYP2D6 alleles which were identified via Sanger sequencing; the SNP identifying the rare alleles were not part of the PHASE analysis. ^2^DropPhase2D6 confirmed linkage on the CYP2D6*21 allele.

**Table 5:** Examination of selected Coriell samples predicted by Ray et al. (2019) to contain novel *CYP2D6* haplotypes.

Sample ID	Genotype	Haplotype ID and SNPs present/absent per Ray et al., 2019 (ref)		Allele linked to “enhancer” SNP
HG00190	*1/*2	H1c	rs5758550, no rs16947, rs5758550, rs35742686 (*3)	*2
HG01067	*2/*4	H1b	rs5758550, no rs16947, rs5758550, rs3892097 (*4), rs1065852 (*10)	*2
NA19672	*1/*68+*2	H2c	rs5758550, rs16947, rs1065852 (*10)	*2
NA19338	*2/*5	H3d	rs16947, no rs5758550, rs5030656 (*9)	*2
HG00651	*5/*36+*10	H1d	rs5758550, no rs16947, rs1065852 (*10)	*5

Extensive genotyping was performed on these Coriell DNA sample to confirm/refute the haplotypes predicted by Ray et al. (2019). Our genotyping and DropPhased2D6 results do not confirm the predicted novel haplotypes.

### DropPhase2D6 Validation With 10X Linked-Reads

The phase of the “enhancer” SNP was determined with 10X Genomics Linked-Reads for 11 Coriell samples (**Table 4**). DropPhase2D6 was successfully performed on one Coriell sample (HG00589) confirming the location of the “enhancer” SNP on the CYP2D6*21 (**Supplemental Figure 1**). 10X technology also revealed that the “enhancer” SNP is on CYP2D6*14, and not the *1 allele of sample NA18552.

## Discussion

In this investigation, we have successfully assigned the “enhancer SNP” to *CYP2D6* star alleles in a data set of >3000 subjects using computational phasing, determined population-specific differences regarding the prevalence of star alleles with and without the “enhancer” SNP, experimentally validated predicted haplotypes using DropPhase2D6 and explored alternative methods for long-distance phasing.

### Computational Phasing

Computational phasing can be utilized to infer CYP2D6-“enhancer” haplotypes present in a population and their frequencies (**Table 2**). Although algorithms, such as PHASE, are powerful statistical tools, results warrant critical evaluation, and ideally, experimental validation. This is especially true for haplotypes that occur at low frequencies as samples homozygous for low frequency events may not be present in the sample set available for analysis. Furthermore, PHASE predictions may be impacted by the differing frequencies of allelic variants among populations (Gaedigk et al., 2017). These limitations are exemplified by CYP2D6*29. When samples of the multi-ethnic dataset (dataset_all) were phased together, 16 of the 129 CYP2D6*29 alleles were predicted to have the “enhancer” SNP. In contrast, when phasing was performed using separate datasets, the European (dataset_E) and African ancestry (dataset_A), PHASE analysis predicted only one CYP2D6*29 allele to carry the “enhancer” SNP. We attribute the observed differences in the phasing results to population-specific haplotype structures. As shown in **Figure 4**, the absence of the “enhancer” SNP on CYP2D6*29 was experimentally confirmed by DropPhase2D6 in eight samples genotyped as compound heterozygotes for the *29 and “enhancer” SNPs (HMW DNA was not available for the other eight samples). Of particular interest is that DropPhase2D6 linked the “enhancer” SNP to the CYP2D6*4 allele of a *4/*29 sample, while PHASE predicted it to be on the *29 allele. This was unexpected, since only one of the 971 CYP2D6*4 alleles was predicted to have the “enhancer” SNP. The CYP2D6*4 allele was sequenced and found to match the *4.004 suballele definition. It remains to be seen, however, whether this suballele always carries the “enhancer” SNP.

Phasing also revealed that the SNPs at positions −1584C>G (rs1080985), 2851C>T (rs16947), and the “enhancer” SNP (rs5758550) are highly linked in some populations, but not others. Therefore, one cannot necessarily assume that each CYP2D6*2 allele carries −1584C>G and the “enhancer” SNP. Also, worth highlighting is that the converse appears to be true for CYP2D6*41, i.e., the vast majority are predicted to lack −1584C>G (rs1080985) and the “enhancer” SNP. Phasing also revealed that a significant number of the CYP2D6*5 gene deletion alleles among the European and African ancestry cohorts have the “enhancer” SNP (9.2 and 9.6%, respectively) and that CYP2D6*1 alleles with the “enhancer” SNP are predominately found among samples with African ancestry (29.2% African and 0.7% European; African and European ancestry samples phased separately, **Table 2**). As shown in **Figure 4**, the “enhancer” SNP was indeed mapped to the CYP2D6*1 allele for 11 samples of African ancestry, while the “enhancer” SNP was on the opposite allele for the samples of European ancestry supporting the computational phasing results. While powerful, computational phasing predictions should always be viewed with caution, especially for complex and highly polymorphic genes such as *CYP2D6*. This notion is highlighted by two samples of African and unknown ancestry (samples 8 and 9, both genotyped as CYP2D6*1/*2), for which phasing predicted the “enhancer” SNP to be on their CYP2D6*2 allele (**Figure 4**). DropPhase2D6, however, experimentally linked the “enhancer” SNP to their *1 allele. One also needs to bear in mind that CYP2D6*1 is a default assignment and therefore comprises alleles with SNPs that were not included in our phasing dataset (e.g., *15, *22, *27, *33, *39, *43, and many other alleles) because these alleles are not routinely genotyped (a small number of samples known to having one of these rare alleles were excluded from the PHASE dataset). Thus, the number of true *1 alleles carrying the “enhancer” SNP is likely lower. Similarly, CYP2D6*11, *12, *19, *21, *59, and numerous other haplotypes were defaulted to a CYP2D6*2 assignment which likely contributes to an over-estimation of true *2 alleles carrying the “enhancer” SNP. A limited number of samples have been genotyped for additional variants revealing, among others, two samples heterozygous for the CYP2D6*59 allele (CYP2D6*1/*59 and *4/*59). DropPhase2D6 unequivocally linked the “enhancer” SNP with the CYP2D6*59 allele. Another two samples for which HMW DNA was available were genotyped as CYP2D6*1/*45; one had the “enhancer” SNP on the *45 allele while the other had it on the *1 allele (**Figure 4**).

Our computational phasing data suggests that the “enhancer” SNP may not, or only in rare cases, be present on certain haplotypes (e.g., CYP2D6*3, *4, *6, *9, *10, *29, and *45) or almost always be present on others (e.g., CYP2D6*35). However, these are predictions and should be viewed with caution. A case in point is CYP2D6*4. We experimentally confirmed the “enhancer” SNP on the CYP2D6*4 allele in one sample; however, this was not the subject predicted by PHASE. Although CYP2D6*4 alleles with the “enhancer” SNP appear to be rare, their frequency may be under-estimated. Thus, it is important to realize that computational phasing, while being a powerful tool, does not necessarily accurately predict on which allele the “enhancer” SNP is located.

### High Molecular Weight DNA Isolation Methods

HMW DNA is a prerequisite for long-distance phasing. To support this study we sought to identify a method that produces DNA of the required integrity from a broad range of biological samples including whole blood, white blood cells and tissue (used fresh or frozen). Of the tested commercially available products, the dialysis-based *MegaLong™ DNA Extraction Kit* yielded consistent results in our hands with superior performance across all positive and negative control reactions. HMW DNA was subjected to DropPhase2D6 within 2 weeks of preparation for best linkage results. Although not systematically evaluated, the integrity of DNA appeared to be compromised over time, due to repeated handling (pipetting) and freeze-thaw cycles.

Since existing and previously handled Coriell DNA samples produced highly variable linkage results or failed altogether, selected DNA samples were re-purchased and handled as described for HMW DNA. Despite these precautions, less than half produced results surpassing the 5% linkage threshold. While all DNAs were of “high quality” (per gel electrophoresis), their integrity was not quite sufficient for long-distance linkage analysis. These challenges could be overcome, however, with a new DNA isolation method the Coriell Institute is planning to offer in the near future.

### DropPhase2D6

Experimentally linking SNPs over distances exceeding those that can be amplified by long-range PCR (up to approximately 20 kb) remains a challenge. Although technologies such as 10X Genomics Linked-Reads (discussed in more detail below) are establishing themselves as powerful tools, cost is a major barrier for their use in routine clinical and research applications and the vendor has recently announced that support for the platform will end in July, 2020. ddPCR is increasingly utilized for DNA and RNA quantification, detection of tumor-derived DNA and pathogen detection to name a few (Li et al., 2018; Liao et al., 2019; Nyaruaba et al., 2019; O'Hara et al., 2019; Oscorbin et al., 2019; Valpione and Campana, 2019), but can also be employed for linkage analysis (Roberts et al., 2014; Karlin-Neumann and Bizouarn, 2018; Lunenburg et al., 2018; Regan and Karlin-Neumann, 2018; Tsujimoto et al., 2018). Since assays can easily be developed by the user and cost-effectively performed, DropPhase2D6 was established to experimentally determine linkage between the “enhancer” SNP and known *CYP2D6* haplotypes. The typical turnaround time including DNA isolation is approximately 24 h.

The interpretation of linkage assay results can be challenging if the majority of DNA molecules do not span the interrogated distance, *i.e.*, the number of droplets producing both FAM and VIC signals (orange cluster) does not exceed or barely exceeds what would be expected by chance. Since there is no standard procedure, we utilized a conservative cut-off value of 5% difference between the percent linkage observed and respective negative controls to call linkage (**Table 3**). Positive calls may be made for samples with differences <5%, especially when all four duplex reactions were performed in triplicate as described by Regan et al. (2015).

DropPhase2D6 results confirmed computational predicted “enhancer” SNP linkage for the majority of samples tested which lends validity to the method and confidence to the results contradicting phase prediction. To further validate DropPhase2D6 we utilized 10X Genomics Linked-Read data of Coriell DNA samples with existing *CYP2D6* genotype data. Although only one of the selected 11 samples was suitable for DropPhase2D6, both methods produced consistent results and the 10X results of the remaining 10 samples were concordant with those predicted by computational phasing (**Table 4**). Once HMW DNA will be available from the Coriell Institute, additional validation will be perform on these samples.

Ray and colleagues have recently published haplotypes containing different combinations of the “enhancer” SNP (rs5758550) and rs16947 (Ray et al., 2019). These tentatively novel haplotypes were inferred from 1000 Genomes data, but have not been verified by genotyping or sequencing by the authors. In order to follow up on those findings as well as determine whether the “enhancer” SNP is indeed located on respective novel haplotypes, five Coriell DNAs were included in the current study. As shown in **Table 5**, none of the predicted haplotypes were consistent with our genotype results and DropPhase data, highlighting the limitations of predicting *CYP2D6* haplotype from 1000 Genomes Project data without experimental confirmation.

One particular limitation for ddPCR-based long-range linkage analyses such as DropPhase2D6 is the availability of HMW DNA, which may prevent re-analysis of samples for which there are no source materials (blood, cells, or tissue) available to prepare DNA of sufficient integrity. The fact that the development of DropPhase2D6 was limited to assays linking rs16947 (present in CYP2D6*2 alleles and many others) or rs3892097 (present in CYP2D6*4) with the “enhancer” SNP (rs5758550) could also be viewed as a limitation. Indeed, linkage could not be established for samples which are homozygous (reference or variant) for rs16947, e.g., samples with a CYP2D6*17/*41, *17/*29, or *1/*9 genotype. However, concordant DropPhase2D6 assay results lends credibility to the approach. We are currently validating additional probe sets that will allow us to analyze samples which have eluded analysis with the probe sets described in this report.

In conclusion, the “enhancer” SNP is not confined to the CYP2D6*2 allele and a given star allele can occur with and without the “enhancer” SNP. Additionally, there are substantial differences among populations in the frequency of alleles with and without the “enhancer” SNP. Thus it remains to be seen, whether the inclusion of this SNP will improve phenotype prediction from genotype data. Furthermore, it has also been proposed that testing the “enhancer” SNP in lieu of SNPs that identify particular star alleles is superior over current genotype approaches. DropPhase2D6 will allow us to map the “enhancer” SNP to star alleles, which is essential for future studies assessing the relationship between *CYP2D6* genotype and activity.

## Data Availability Statement

All datasets generated for this study are included in the article/**Supplementary Material**.

## Ethics Statement

The studies involving human participants were reviewed and approved by the Children's Mercy Hospital Internal Review Board. Written informed consent to participate in this study was provided by the participants.

## Author Contributions

AG and SL conceived the study. AG, EB, and RG designed the study and assays. EB and WW conducted experimental work. AG, EB, RG, NM, and WW analyzed data. MC and AB contributed materials and data. AG, EB, WW, RG, and NM wrote the manuscript. All other authors provided critical feedback.

## Funding

This work was supported by the Eunice Kennedy Shriver National Institute of Child Health and Human Development grant U54 HD090258-01. The Liver Tissue Cell Distribution System is funded by the National Institutes of Health [Contract N01-DK-7-0004/HHSN267200700004C]. The project, entitled “Laboratory of Developmental Biology,” was supported by an award from the Eunice Kennedy Shriver National Institute of Child Health & Human Development of the National Institutes of Health [Award 5R24HD0008836].

## Conflict of Interest

The authors declare that the research was conducted in the absence of any commercial or financial relationships that could be construed as a potential conflict of interest.

## Supplementary Material

Table S1 Genotyping methods. To standardize our genotyping method we utilized a reporting format that was recommended by PharmVar and PharmGKB (Nofziger et al., 2020). The first tab labeled ‘Methods’ provides brief overviews of the methods used for genotyping. The second tab labeled ‘CYP2D6 SNPs tested’ provides detailed information of sequence variants tested. All SNP positions are in reference to the *CYP2D6* RefSeq NG_008376.3 with the ATG start codon being +1 according to PharmVar at https://www.pharmvar.org. ^1^ rs133333 is in complete linkage with rs5758550. Initially, rs133333 and rs5758550 were both reported as possible “enhancer” SNPs and genotyping was done for rs133333, rs5758550 or both. Ultimately rs5758550 was characterized as the functional “enhancer” SNP. ^2^ SNP utilized for computational PHASE analysis. ^3^ Samples known to have these SNPs were excluded from the computational PHASE dataset; however, some of these samples were chosen for the DropPhase2D6 assay if HMW gDNA was available.

Figure S1 Visualization of 10X Genomics Linked-Reads. The genotype of Coriell sample HG00589 was determined by genotyping and Sanger sequencing and the “enhancer” SNP linked to the *CYP2D6*21* allele by DropPhase2D6. 10X Genomics Linked-Reads analysis corroborated the DropPhase result. As can be seen in the Loupe screenshots, the “enhancer” SNP is located on the same chromosome (arrow, upper line representing chromosome 1) as the *CYP2D6*21* core allele SNPs rs16947 and rs72549352 (arrows, also on the upper line). The top panel indicates Chr22 coordinates and the blue graph at the bottom represents the genes in which the SNPs are located. Additional details can be found at https://www.10xgenomics.com.
