# Supplementary figures and images for: Long-Distance Phasing of a Tentative “Enhancer” Single-Nucleotide Polymorphism With CYP2D6 Star Allele Definitions
Source: Front Pharmacol. 2020 May 8;11:486. doi: 10.3389/fphar.2020.00486 (PMC7226225; doi:10.3389/fphar.2020.00486)

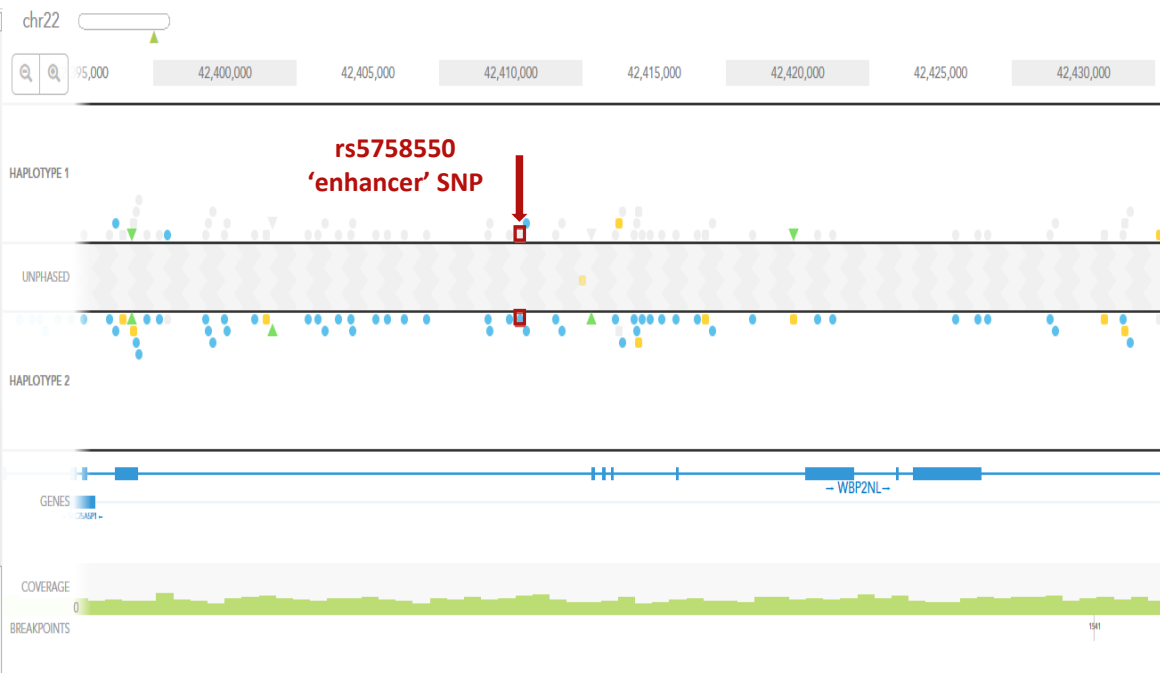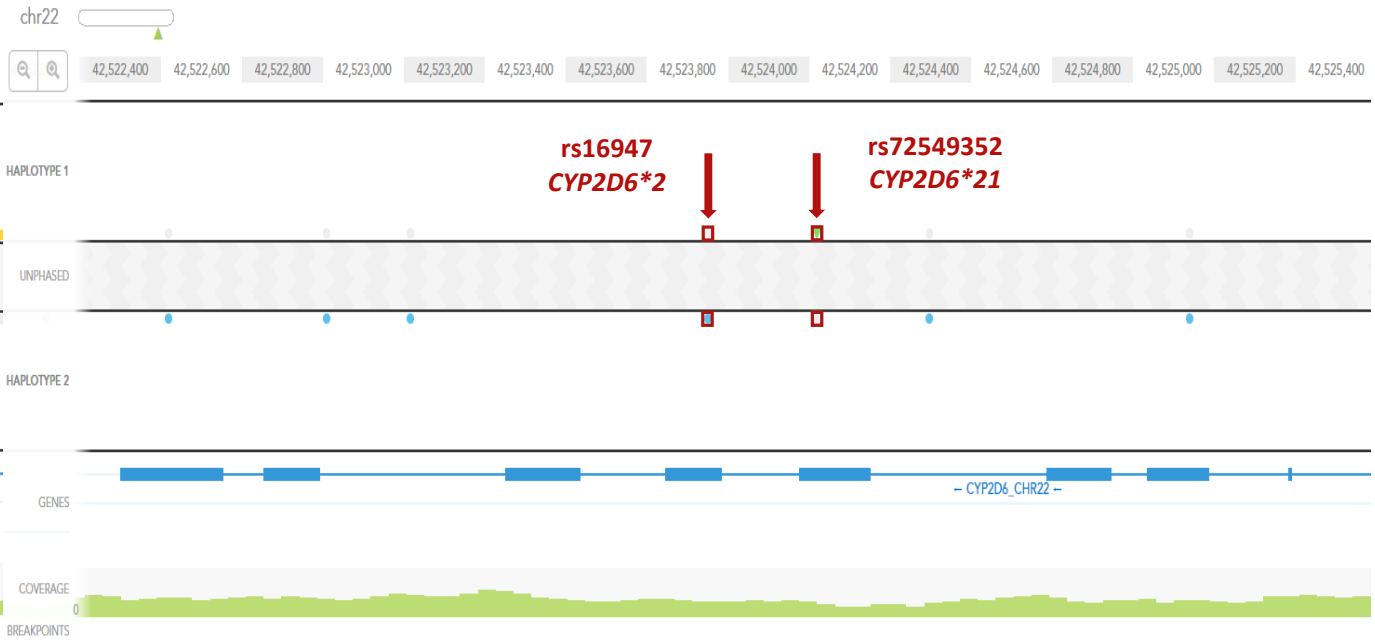

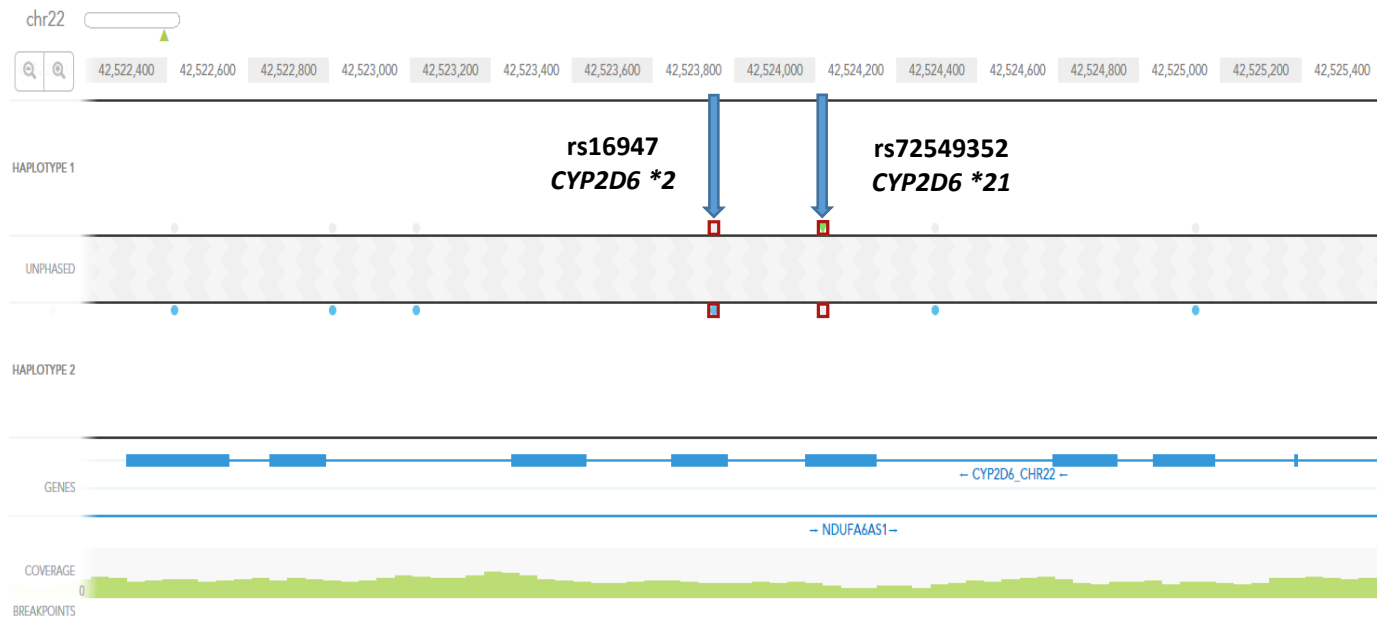

Supplement: Figure S1 — Visualization of 10X Genomics Linked-Reads. The genotype of Coriell sample HG00589 was determined by genotyping and Sanger sequencing and the “enhancer” SNP linked to the CYP2D6*21 allele by DropPhase2D6. 10X Genomics Linked-Reads analysis corroborated the DropPhase result. As can be seen in the Loupe screenshots, the “enhancer” SNP is located on the same chromosome (arrow, upper line representing chromosome 1) as the CYP2D6*21 core allele SNPs rs16947 and rs72549352 (arrows, also on the upper line). The top panel indicates Chr22 coordinates and the blue graph at the bottom represents the genes in which the SNPs are located. Additional details can be found at https://www.10xgenomics.com. [file Image_1.pdf]
